# Interfacial Bond Strength of CAD/CAM Resin Composites on Dentin vs. Composite Substrates: Influence of Dual-Cure and Self-Adhesive Resin Cements

**DOI:** 10.3390/polym18020224

**Published:** 2026-01-15

**Authors:** Oyun-Erdene Batgerel, Oktay Yazıcıoğlu, Emine Kıtın, Burç İhsan Gençel, Fatih Yamak, Süreyya Ergün Bozdağ, Rafat Sasany

**Affiliations:** 1 Department of Restorative Dentistry, Biruni University, Merkezefendi Mahallesi, G/75 Sokak, 1-1 (M.G.), Zeytinburnu, Istanbul 34015, Turkey; oerdene@biruni.edu.tr; 2 Department of Restorative Dentistry, İstanbul University, Beyazıt Square (Beyazıt Meydanı) Fatih, Istanbul 34126, Turkey; oktayy@istanbul.edu.tr; 3 Department of Restorative Dentistry, Istanbul Health and Technology University, Sütlüce Mah., İmrahor Cad., 82, Beyoğlu, Istanbul 34445, Turkey; eminekitin@istanbul.edu.tr; 4 Vocational School of Health Services, Department of Dentistry Services, Istanbul University, Cerrahpaşa Mahallesi Kocamustafapaşa Caddesi, 53, Fatih, Istanbul 34098, Turkey; bgencel@istanbul.edu.tr; 5 Department of Mechanical Engineering, İstanbul Technical University, Ayazağa Campus Reşitpaşa Mahallesi Sarıyer, Istanbul 34469, Turkey; yamakf@itu.edu.tr (F.Y.); bozdager@itu.edu.tr (S.E.B.); 6 Department of Prosthodontics, Faculty of Dentistry, Biruni University, Merkezefendi Mahallesi, G/75 Sokak, 1-13 (M.G.), Zeytinburnu, Istanbul 34015, Turkey

**Keywords:** composite, interfacial bond strength, self-adhesive resin cements

## Abstract

This in vitro study evaluated the shear bond strength (SBS) of four CAD/CAM (Computer aided design/Computer aided manufacturing) polymer-based indirect composites bonded to dentin and microhybrid composite substrates using two resin cements. Gradia Plus (GP), Ceramage (Ce), Tescera ATL (TA), and Lava Ultimate (LA) were fabricated into cylindrical specimens (3 × 3 mm). Dentin substrates were obtained from extracted molars, while composite substrates were prepared from Filtek Z250 (4 mm × 2 mm). Bonding was performed using either a self-adhesive resin cement (RelyX U200; RU200) or a dual-cure adhesive resin cement (RelyX Ultimate; RU), resulting in 16 experimental groups (n = 12 per group). SBS was measured using a universal testing machine (1 mm/min), and failure modes were assessed under stereomicroscopy. Bond strength was significantly higher on composite substrates than on dentin (*p* < 0.001), primarily due to favorable polymer–polymer compatibility and matrix interdiffusion, which improved stress accommodation at the adhesive interface. TA and Ce showed superior adhesion when combined with RU, while LA exhibited the lowest values, particularly on dentin bonded with RU200. Overall, the dual-cure adhesive system provided stronger bonding than the self-adhesive system (*p* < 0.05). These findings highlight the influence of substrate type, composite architecture, and cement chemistry on interfacial performance in indirect polymer-based restorations.

## 1. Introduction

Minimally invasive dentistry emphasizes preserving natural tooth structure and function while minimizing damage to hard tissues and the pulp [1]. The performance of polymer-based restorative materials is largely governed by their intrinsic network structure, filler morphology, and the quality of the adhesive interphase formed at the restoration–substrate interface. In indirect composites, the polymerization process is conducted under controlled laboratory conditions involving heat, pressure, and high-intensity light, which increases monomer conversion, cross-link density, and network homogeneity compared to chairside polymerized direct composites [1,2,3]. These controlled curing environments reduce polymerization shrinkage and residual stress, factors that can otherwise impair interfacial adhesion and dimensional stability in restorative polymers [4,5]. Lower residual stress may further decrease stress transfer to the adhesive layer during shear loading, thereby reducing interfacial stress concentration and improving resistance to debonding [4,5].

Modern CAD/CAM resin composites are industrially polymerized, highly cross-linked dimethacrylate-based materials (e.g., Bis-GMA, UDMA, Bis-EMA, and/or TEGDMA) reinforced with silanized submicron ceramic or glass fillers. Differences in matrix–filler architecture (e.g., nanoceramic versus highly filled laboratory composites) and in filler characteristics (type, size distribution, and volume fraction) may alter surface energy and wettability, as well as micromechanical interlocking and adaptation with resin cements, ultimately contributing to material-dependent variations in shear bond strength [4,5,6,7,8,9,10].

Clinically, CAD/CAM resin composite blocks are indicated for laminate veneers, inlays/onlays/overlays, endocrowns, and partial-coverage posterior restorations, especially when conservative tooth preparation is desired [6,7,8]. Compared with conventionally fabricated indirect composites and chairside direct composites, CAD/CAM blocks offer standardized polymerization and microstructural homogeneity, which may translate into more consistent mechanical behavior and cementation performance [8,9,10]. Notably, filler type and volume fraction remain key determinants of hardness, wear resistance, and crack-propagation resistance, thereby influencing long-term clinical performance [11,12].

Optimized laboratory polymerization can improve the modulus and microstructural integrity by enhancing polymerization kinetics and limiting oxygen inhibition, which may support long-term stability [13,14]. However, clinical longevity remains strongly dependent on the integrity of the adhesive interphase, particularly on dentin, where hydrated collagen, smear-layer remnants, and outward dentinal fluid flow can restrict monomer infiltration and compromise micromechanical interlocking [15,16]. Moreover, intrinsic substrate variability (tubule density and orientation, mineralization, and baseline moisture) can generate interfacial heterogeneity and bonding scatter even when surface preparation is standardized [15,16]. By contrast, bonding to composite substrates is often more predictable because polymer–polymer interactions can facilitate monomer diffusion and limited swelling of the existing resin matrix, promoting a more integrated interphase and improved energy dissipation under load [17,18].

Resin cement chemistry plays a central role in interfacial performance. Self-adhesive resin cements rely on acidic functional monomers to simultaneously demineralize and infiltrate the substrate; however, their limited smear-layer modification and reduced chemical coupling can result in weaker bonding, particularly on dentin. Importantly, the lower performance of self-adhesive cements may be attributed to limited chemical interaction between acidic functional monomers and the mineral phase of dentin, which may be insufficient to form a stable, continuous hybrid layer and a durable adhesive interphase [15,16,19]. Dual-cure adhesive cements, when used with separate universal primers, provide enhanced hybridization, better wettability of the substrate, and increased polymerization depth, which collectively contribute to higher bond strength and interphase continuity [20,21,22].

Despite the widespread use of CAD/CAM resin composites, limited data exist comparing their interfacial performance under different substrate conditions using adhesive systems with distinct polymerization and bonding mechanisms. Understanding how composite architecture, cement chemistry, and substrate type influence shear bond strength (SBS) is essential to optimize adhesive protocols for polymer-based restorative applications.

Therefore, this study aimed to evaluate the shear bond strength of different CAD/CAM indirect resin composites bonded to dentin and microhybrid composite substrates using self-adhesive and dual-cure adhesive cement systems. The null hypothesis stated that SBS would not be affected by (1) composite material type, (2) resin cement system, or (3) substrate type. Although the factors were evaluated as independent variables, potential interaction effects were anticipated, whereby cement performance could depend on the specific CAD/CAM composite chemistry and differ between dentin and composite substrates.

## 2. Materials and Methods

### 2.1. Ethical Standards

The study protocols were conducted in accordance with ISO/TS 11405 guidelines for bond strength testing. All procedures complied with the ethical principles of the Declaration of Helsinki (2013). Extracted teeth were obtained after written informed consent, anonymized, and used solely for laboratory research purposes. No clinical intervention was performed in this in vitro study.

### 2.2. Preparation of Dentin Substrates

Forty-eight sound human molars extracted for clinical reasons unrelated to this study were selected. Teeth exhibiting caries, restorations, endodontic treatment, or fractures were excluded. Residual soft tissues were mechanically removed, and specimens were stored in 0.1% thymol solution at 4 °C for a maximum of six months to inhibit microbial activity without chemically altering the dentin substrate.

Each tooth was embedded in autopolymerizing acrylic resin using pre-fabricated silicone molds. The coronal portion was sectioned 2–3 mm apical to the cementoenamel junction using a low-speed, water-cooled diamond saw (Isomet 1000, Buehler, Lake Bluff, IL, USA). To standardize the surface morphology and smear layer, exposed dentin was abraded for 60 s under continuous irrigation using 600-grit silicon carbide paper. This produced a homogeneous substrate suitable for adhesive interphase formation.

### 2.3. Composite Substrate Preparation

Composite substrates were fabricated as 48 standardized discs (4 mm diameter × 2 mm thickness) using a microhybrid composite resin (Filtek Z250, 3M ESPE, St. Paul, MN, USA). The discs were produced in silicone molds to ensure dimensional accuracy. Resin was inserted incrementally in 1 mm layers to minimize polymerization stress and thermal gradients. Each increment was polymerized for 20 s using an LED curing device (Demi Ultra, Kerr, Orange, CA, USA) delivering 1100 mW/cm^2^ irradiance.

To reduce oxygen inhibition and suppress surface radical quenching, a transparent polyester strip was applied over the final increment during curing. All composite discs were stored in light-proof containers at room temperature for 24 h to complete post-cure polymerization.

### 2.4. Indirect Composite Specimens

Four indirect restorative composites were evaluated: Lava Ultimate (LA) is a nanoceramic CAD/CAM resin composite based on a cross-linked dimethacrylate matrix (Bis-GMA/TEGDMA) reinforced with silica and zirconia nanofillers. Gradia Plus (GP) is a laboratory composite containing a dimethacrylate resin matrix (Bis-GMA, UDMA, and TEGDMA) with ceramic fillers. Tescera ATL (TA) is a highly filled laboratory composite based on Bis-GMA/TEGDMA and reinforced with amorphous silica fillers. Ceramage (Ce) is a UDMA-based laboratory composite reinforced with zirconium silicate fillers. Detailed material compositions are provided in Table 1.

Lava Ultimate (LA): Nanoceramic polymer blocks (3M ESPE, St. Paul, MN, USA) were designed using AutoCAD 2026 software, milled via CAM, and sectioned with a water-cooled diamond saw (Isomet 1000).

Gradia Plus (GP): Incrementally layered in 1 mm segments, each polymerized for 10 s using GC Steplight SL-1, followed by post-curing for 3 min in a GC Labolight LV-III chamber.

Tescera ATL (TA): Composite layers were polymerized under pressure for 2 min in the Tescera Light Cup unit and then subjected to pressure–heat–light polymerization for 10–13 min in the Tescera curing unit.

Ceramage (Ce): Layers were polymerized for 20 s per increment with Sublite, followed by 5 min post-curing using Solidilite.

### 2.5. Cementation Procedures

Bonding was standardized using a universal bonding device (UltraTester™, Ultradent, South Jordan, UT, USA), ensuring uniform compressive force and perpendicular specimen alignment. Dentin substrates (DS) and composite substrates (MS) mounted in acrylic blocks were positioned in the apparatus.

A load of 500 ± 50 g was applied for 10 min to stabilize the adhesive interphase under constant pressure. This controlled seating load was selected to simulate standardized clinical seating pressure; however, the uniform load application may favor cements with lower viscosity and improved flow during the initial setting phase. To suppress oxygen inhibition at the bonding margins, a glycerin gel (Liquid Strip, Ivoclar Vivadent, Liechtenstein) was applied prior to light curing and rinsed after polymerization. This procedure was identical across all cementation groups [23].

#### 2.5.1. Self-Adhesive Cement (RU200)

RelyX U200 (3M ESPE) was applied directly to substrates without additional surface pretreatment, following manufacturer guidelines. Polymerization occurred via LED activation for 20 s.

#### 2.5.2. Dual-Cure Adhesive Cement (RU)

RelyX Ultimate (3M ESPE) was used in combination with a universal adhesive system. Substrates were etched for 20 s using Scotchbond Universal Etchant, followed by application and light curing of Single Bond Universal. Cement was then applied and polymerized according to manufacturer protocol [24].

### 2.6. Experimental Design

**Figure 1:**
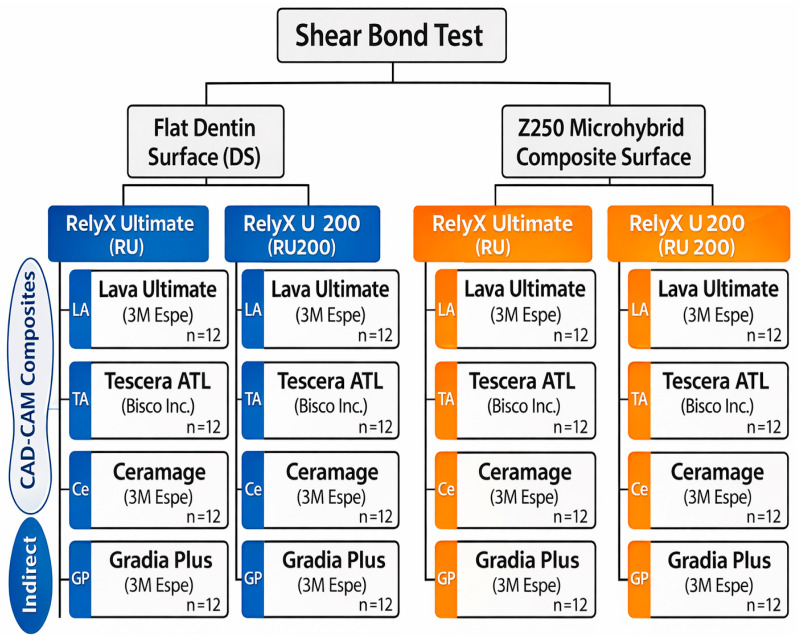
Schematic illustration of the experimental workflow, including substrate preparation (dentin and composite), fabrication of indirect CAD/CAM resin composite specimens, cementation protocols using self-adhesive and dual-cure resin cements, shear bond strength testing, and failure mode analysis.

**Figure 2:**
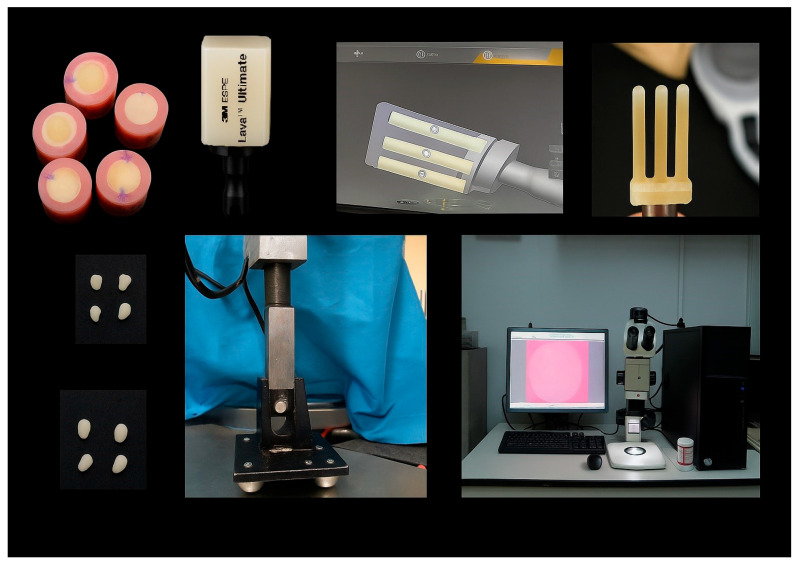
Preparation of indirect resin composite specimens, standardized adhesive cementation onto dentin and composite substrates using a universal bonding device, and shear bond strength testing configuration showing the direction of applied load.

A three-factor experimental design was adopted, including restorative material (four levels), cement type (two levels), and substrate type (two levels). This resulted in 16 experimental groups (n = 12 per group). The workflow and group allocation are shown in Figure 1 and Figure 2.

### 2.7. Shear Bond Strength Testing

Shear bond strength (SBS) was assessed using a universal testing machine (MTS Mini-ics Model 858, MTS, Eden Prairie, MN, USA). A custom loading jig applied force parallel to the adhesive interface at a crosshead speed of 1 mm/min until debonding. Maximum load (N) was recorded. SBS (MPa) was calculated by dividing the failure load by the bonded area (mm^2^).

### 2.8. Failure Mode Analysis

**Figure 3:**
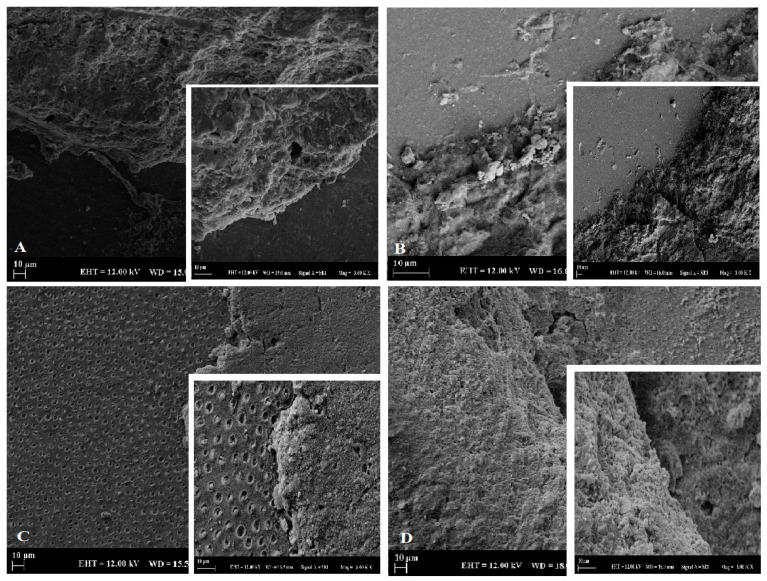
Representative SEM images showing interfacial failure characteristics of indirect resin composites bonded to dentin and composite substrates. Arrows indicate interfacial gaps or cohesive fracture zones. GP bonded to dentin using RelyX Ultimate (**A**); Ce bonded to dentin using RelyX Ultimate (**B**); TA bonded to composite substrate using RelyX U200 (**C**); LA bonded to composite substrate using RelyX Ultimate (**D**). Main images acquired at ×1000 magnification; insets at ×3000 magnification. Scale bar = 10 µm.

Post-fracture samples were examined under stereomicroscopy (Edmund Optics, Barrington, NJ, USA; ×32 magnification). Failure patterns were classified as adhesive (interfacial), cohesive (within cement or substrate), or mixed. Attention was given to identifying crack origin zones and interphase disruptions. Representative images and classifications are presented in Figure 3.

### 2.9. SEM Characterization

Representative fractured specimens were gold/palladium-sputter-coated and analyzed using SEM (Zeiss EVO LS 10, Carl Zeiss, Oberkochen, Germany). Surface features, filler exposure, and interfacial morphology were examined at ×1000 and ×3000 magnifications.

### 2.10. Statistical Analysis

Normality of SBS data was tested using the Shapiro–Wilk method. Due to non-parametric distribution, Kruskal–Wallis tests were used for group comparisons, followed by Mann–Whitney U tests with Holm correction for multiple comparisons. Failure mode distributions were compared using Pearson’s χ^2^ test. Statistical significance was set at α = 0.05.

## 3. Results

### 3.1. Comparison Between Dentin and Composite Substrates

The Mann–Whitney U test showed a statistically significant difference in SBS between the dentin (DS) and composite (MS) substrates (U = 812.00, Z = −4.68, *p* < 0.001). Mean SBS values were significantly higher for composite substrates (9.3 ± 3.5 MPa) compared with dentin substrates (5.6 ± 2.9 MPa)**,** indicating higher bond strength values on composite specimens (Table 2).

### 3.2. Comparison Among Restorative Materials

SBS values differed significantly among the tested restorative materials within each cement–substrate combination (*p* < 0.001). The lowest SBS value was recorded for the LA/DS/RU200 group (3.02 ± 1.34 MPa), whereas the highest value was observed in the TA/MS/RU group (13.17 ± 2.45 MPa).

When bonded to composite substrates using the dual-cure adhesive cement (RU), TA and Ce exhibited the highest SBS values, followed by GP and LA. In contrast, for dentin substrates bonded with the self-adhesive cement (RU200), SBS values decreased in the order TA > Ce > GP > LA.

### 3.3. Comparison Between Resin Cements

A statistically significant difference was detected between cements when bonded to dentin (U = 660.00, Z = −3.91, *p* < 0.001), with RelyX Ultimate (RU) producing higher SBS values than RelyX U200 (RU200). Conversely, no significant difference was detected between the two cements when bonded to composite substrates (U = 1248.00, Z = −0.87, *p* = 0.385).

### 3.4. Failure Mode Distribution

Failure mode analysis revealed distinct patterns depending on substrate type (Table 3). Dentin specimens predominantly exhibited adhesive failures (66.7%), followed by mixed (25.0%) and cohesive failures (8.3%). In contrast, composite substrate specimens showed a higher incidence of mixed (37.5%) and cohesive failures (41.7%), with fewer adhesive failures (20.8%).

### 3.5. SEM Observations

Specimens bonded with the self-adhesive cement (RU200) frequently exhibited interfacial gaps and discontinuities, particularly at the dentin–cement interface. In contrast, specimens bonded with the dual-cure adhesive cement (RU) showed more continuous and homogeneous cement–substrate interfaces, especially in the TA and Ce groups. These microscopic findings were consistent with the higher SBS values and increased cohesive failure patterns observed in composite substrate groups. Representative SEM images are shown in Figure 3.

## 4. Discussion

The null hypothesis of this study, which stated that no significant differences would be found in shear bond strength among the tested CAD/CAM composite materials and adhesive systems on dentin and composite substrates, was rejected. The results demonstrated that all three factors significantly affected interfacial bonding performance, highlighting the complex interaction between material composition, adhesive chemistry, and substrate characteristics.

One of the principal findings of this study was that bonding to composite substrates resulted in significantly higher SBS values than bonding to dentin, regardless of the resin cement used. This outcome can be attributed to favorable polymer–polymer interactions between the resin cement and the microhybrid composite substrate [21,22,23,24,25,26]. Unlike dentin, which is a hydrated, heterogeneous biological tissue, composite substrates provide a chemically compatible and relatively homogeneous surface, facilitating monomer diffusion, partial swelling of the resin matrix, and improved micromechanical interlocking [26]. These findings are consistent with previous reports indicating enhanced bonding effectiveness at composite–resin interfaces compared with polymer–dentin interfaces [27,28].

Among the tested indirect restorative materials, Tescera ATL exhibited the highest SBS values, particularly when bonded to composite substrates using the dual-cure adhesive cement. This superior performance may be explained by its high filler content and multi-stage polymerization protocol involving light, heat, and pressure, which promotes increased monomer conversion, improved cross-link density, and enhanced polymer network homogeneity. Differences in degree of conversion and network stiffness may also affect cement adaptation and interfacial connectivity by influencing surface reactivity and the ability of resin cements to wet and conform to microstructural features [27,28]. In contrast, Lava Ultimate consistently demonstrated the lowest SBS values across both substrates and cement systems. The highly prepolymerized resin matrix and high nanoceramic filler fraction of Lava Ultimate likely limit the availability of unreacted functional groups and reduce chain mobility, thereby restricting chemical interaction and interdiffusion with resin cements [19,29,30].

The type of resin cement also played a decisive role in bonding performance. The dual-cure adhesive resin cement (RelyX Ultimate) produced significantly higher SBS values than the self-adhesive cement (RelyX U200), particularly on dentin substrates. This finding can be attributed to the use of an etch-and-rinse adhesive strategy in combination with the dual-cure cement, which enables effective smear-layer removal, resin infiltration into demineralized dentin, and formation of a more stable hybrid layer [31,32]. In contrast, self-adhesive resin cements rely on acidic functional monomers for simultaneous demineralization and infiltration; however, their limited interaction with the smear layer may compromise bonding effectiveness on dentin [33]. In this context, adhesive application and hybrid-layer formation are major contributors to bond strength in the dual-cure system, whereas the resin cement mainly acts as a luting material that supports load transfer and stress distribution across the interface. Failure mode analysis further supported these findings. Adhesive failures predominated in dentin specimens, indicating weaker interfacial bonding and limited energy dissipation at the dentin–cement interface. In addition, the shear testing configuration may induce non-uniform stress concentration at the adhesive interface, which can further promote adhesive debonding in dentin groups. Conversely, composite substrate groups exhibited higher proportions of mixed and cohesive failures, suggesting stronger interfacial continuity and improved stress distribution within the bonded assembly. These observations were corroborated by SEM analysis, which revealed interfacial gaps and discontinuities in self-adhesive cement groups, which may be related to reduced wetting/adaptation, polymerization-shrinkage-related interfacial stress, and/or diminished chemical interaction (particularly on dentin) in self-adhesive systems, whereas dual-cure adhesive cement groups showed more continuous and homogeneous interfaces, particularly in specimens bonded to composite substrates.

The composite substrate model used in this study may also be considered a laboratory simulation of a deep margin elevation (DME) scenario. The results indicated that the presence of a composite substrate did not compromise bonding performance; instead, SBS values were enhanced when compatible materials and adhesive strategies were employed. This finding aligns with previous studies suggesting that DME does not adversely affect adhesive performance and may represent a conservative clinical approach when restoration margins extend subgingivally [34,35,36,37,38].

From a clinical perspective, achieving sufficient bond strength is essential for the long-term success of indirect restorations [39]. Only specimens bonded with the dual-cure adhesive cement approached SBS values commonly considered acceptable for durable indirect restorations, emphasizing the importance of appropriate cement selection and surface conditioning protocols. While lower SBS values observed in certain material combinations may still be acceptable for provisional or low-load applications, they may be less suitable for long-term restorations subjected to high occlusal forces.

Several limitations of this study should be acknowledged. Surface pretreatments (e.g., air abrasion, silanization, or laser conditioning) were intentionally excluded to isolate the effects of material type, substrate, and cement system; accordingly, the findings may not fully reflect clinically optimized bonding protocols. Likewise, artificial aging procedures such as thermocycling and mechanical fatigue were not performed, which limits extrapolation to long-term clinical performance. Although thymol storage is widely used for preserving extracted teeth, prolonged storage may subtly alter dentin surface chemistry and/or collagen integrity, potentially affecting adhesive monomer diffusion and interfacial infiltration. Moreover, the use of freshly polymerized composite substrates represents an idealized bonding condition and may differ from clinically aged composite restorations, where hydrolytic degradation and reduced surface reactivity could diminish bonding effectiveness. Finally, as an in vitro investigation, this study cannot replicate the full complexity of intraoral conditions. Future work should incorporate standardized surface conditioning, artificial aging, and clinically relevant loading protocols to better clarify the durability of these adhesive interfaces.

## 5. Conclusions

Within the limitations of this in vitro study, the following conclusions can be drawn:

1. The dual-cure adhesive resin cement (RelyX Ultimate), used in combination with an etch-and-rinse adhesive strategy, produced significantly higher shear bond strength values than the self-adhesive resin cement (RelyX U200), particularly when bonded to dentin substrates. This finding underscores the importance of effective surface conditioning and hybrid-layer formation for achieving reliable adhesion to hydrated dentin.
2. Bonding to composite substrates resulted in significantly higher shear bond strength values than bonding to dentin, regardless of the resin cement used. This outcome highlights favorable polymer–polymer interactions at composite resin interfaces and supports the use of composite substrates in restorative scenarios such as deep margin elevation.
3. Among the indirect CAD/CAM resin composites evaluated, Tescera ATL exhibited the highest bond strength values, likely due to its high filler content and multi-stage polymerization protocol involving light, heat, and pressure, which enhance polymer network homogeneity and interfacial compatibility.
4. Lava Ultimate consistently demonstrated the lowest bond strength values across substrates and cement systems. Its highly prepolymerized resin matrix and high nanoceramic filler fraction may limit chain mobility and reduce chemical interaction with resin cements, thereby compromising interfacial bonding effectiveness.
5. Failure mode and SEM analyses corroborate the mechanical findings, revealing predominantly adhesive failures on dentin substrates and a higher incidence of mixed and cohesive failures on composite substrates, indicative of stronger and more continuous adhesive interfaces.

## Figures and Tables

**Table 1:** Materials, resin matrix composition, filler characteristics, and manufacturers of all products used in the study.

Composite Resins	Main Matrix Monomers	Components	Manufacturer
**Gradia Plus**	1–5% Bis-GMA, 5–10% TEGDMA, 1–5% UDMA	Ceramic particles	GC Corp., Tokyo, Japan
**Ceramage**	5–10% UDMA	Zirconium silicate, 73%	Shofu Inc., Kyoto, Japan
**Tescera ATL**	TEGDMA, Bis-GMA	Amorphous silica, 85%	Bisco Inc., Schaumburg, IL, USA
**LAVA Ultimate**	80% nanoceramic composite Bis-GMA/TEGDMA	Silica and zirconia fillers	3M ESPE, St. Paul, MN, USA
**Scotchbond Universal Etchant**	Phosphoric acid	35% phosphoric acid	3M ESPE, St. Paul, MN, USA
**Z250**	Organic matrix: Bis-GMA, UDMA, Bis-EMA	Zirconia/silica filler (0.01–3.5 μm, 82% wt-60% vol)	3M ESPE, St. Paul, MN, USA
**Single Bond Universal**	10MDP, phosphate monomers, dimethacrylate resins, HEMA, methacrylate-modified, polyalkenoic acid copolymer, filler, ethanol, water, initiator, silane		3M ESPE, St. Paul, MN, USA
**RelyX Ultimate** Universal adhesive cement	Methacrylate monomers, silanized fillers, initiators, stabilizers, dual-cure activators		3M ESPE, St. Paul, MN, USA
**RelyX U200** Self-adhesive resin cement	Matrix: hydroxyethylmethacrylate (HEMA), Bis-GMA; fillers: fluoro alumino silicate glass, zirconium silica		3M ESPE, St. Paul, MN, USA
**Single Bond Universal**	Universal adhesive; 10-MDP, HEMA, dimethacrylate resins, silane, ethanol, water		3M ESPE, St. Paul, MN, USA
**Scotchbond Universal Etchant**	35% phosphoric acid etchant		3M ESPE, St. Paul, MN, USA
**Thymol solution (0.1%)**	Storage medium for extracted teeth		Sigma-Aldrich, St. Louis, MO, USA
**Autopolymerizing acrylic resin**	Specimen embedding material		Vertex Dental, Zeist, The Netherlands
**Silicone molds**	Specimen fabrication molds		Zhermack, Badia Polesine, Italy
**Polyester strip**	Oxygen inhibition control during curing		GC Corp., Tokyo, Japan
**Glycerin gel (Liquid Strip)**	Oxygen inhibition barrier during cementation		Ivoclar Vivadent, Schaan, Liechtenstein

**Table 2:** Shear bond strengths of CAD/CAM composites (MPa): mean, standard deviation, (minimum, maximum), and [median].

Groups	RU		RU200	
	DS	MS	DS	MS
LA	3.02 ± 1.34 ^d^ (0.85–4.35) [3.51]	4.67 ± 1.04 ^cd^ (2.15–7.20) [5.5]	5.69 ± 2.69 ^cd^ (3.19–13.99) [3.51]	5.47 ± 1.59 ^cd^ (3.03–7.76) [5.44]
TA	4.81 ± 1.63 ^c^ (1.88–7.47) [5.35]	12.59 ± 1.14 ^a^ (10.04–15.74) [12.15]	8.05 ± 3.68 ^b^ (1.57–15.28) [8.05]	13.17 ± 2.45 ^a^ (9.52–16.53) [12.93]
Ce	3.30 ± 1.35 ^d^ (2.16–6.34) [2.98]	8.21 ± 1.37 ^b^ (5.43–13.44) [9.66]	6.98 ± 2.33 ^bc^ (5.35–12.45) [8.12]	8.63 ± 1.93 ^ab^ (8.01–14.85) [10.45]
GP	3.62 ± 1.23 ^d^ (1.30–5.79) [4.7]	9.71 ± 2.97 ^b^ (6.12–10.90) [7.85]	8.63 ± 2.13 ^b^ (3.66–12.39) [6.77]	10.88 ± 2.12 ^ab^ (8.21–13.37) [8.94]

Different lowercase letters indicate statistically significant differences among groups (*p* < 0.05).

**Table 3:** Evaluation of fracture types.

Substrate	Adhesive n (%)	Mixed n (%)	Cohesive n (%)
DS	16 (66.7%)	6 (25.0%)	2 (8.3%)
MS	5 (20.8%)	9 (37.5%)	10 (41.7%)

The distribution of failure modes differed significantly between dentin and composite substrates (χ^2^ test, *p* < 0.05).

## Data Availability

The data supporting the findings of this study are available from the corresponding author upon reasonable request.

## Abbreviations

The following abbreviations are used in this manuscript:

CAD/CAM	Computer-Aided Design/Computer-Aided Manufacturing
SBS	Shear Bond Strength
RU	RelyX Ultimate
RU200	RelyX U200
TA	Tescera ATL
Ce	Ceramage
GP	Gradia Plus
LA	Lava Ultimate
SEM	Scanning Electron Microscopy
MS	Composite Substrate
DS	Dentin Substrate
Bis-GMA	Bisphenol A-Glycidyl Methacrylate
UDMA	Urethane Dimethacrylate
Bis-EMA	Ethoxylated Bisphenol A Dimethacrylate
TEGDMA	Triethylene Glycol Dimethacrylate
